# Assessment of the Technological Quality of X5CRNI18-10 Steel Parts after Laser and Abrasive Water Jet Cutting Using Synthetic Index of Technological Quality

**DOI:** 10.3390/ma14174801

**Published:** 2021-08-24

**Authors:** Marcin Romanowski, Czesław Łukianowicz, Marzena Sutowska, Wojciech Zawadka, Danil Yurievich Pimenov, Krzysztof Nadolny

**Affiliations:** 1 Department of Production Engineering, Faculty of Mechanical Engineering, Koszalin University of Technology, Racławicka 15-17, 75-620 Koszalin, Poland; marcinro@wp.pl (M.R.); czeslaw.lukianowicz@tu.koszalin.pl (C.Ł.); marzena.sutowska@tu.koszalin.pl (M.S.); wojciech.zawadka@tu.koszalin.pl (W.Z.); 2 Department of Automated Mechanical Engineering, South Ural State University, Lenin Prosp.76, 454080 Chelyabinsk, Russia; danil_u@rambler.ru

**Keywords:** laser cutting, water jet cutting, technological quality, quality assessment, X5CRNI18-10 steel

## Abstract

Despite extensive knowledge of the cutting methods described, no universal method has been developed so far for evaluating the technological quality of elements shaped by different cutting processes. The aim of the research described in this article was to fill this gap and to propose the author’s methodology for the assessment of the technological quality of the surface of X5CRNI18-10 steel shaped as a result of laser cutting and abrasive water jet cutting. A synthetic index of technological quality assessment of the surface after cutting *CTQ* (cutting technological quality) was proposed. Three groups of factors were taken into account in the assessment of technological quality of the surface after cutting: selected surface texture parameters (arithmetic mean deviation of the surface *Sa* and total height of the surface *St*), results of measurements of dimensional accuracy of cut elements (length deviation *LD* and width deviation *WD*) as well as indicators of surface morphology estimated on the basis of microscopic images of the surface after cutting (deformation intensity *DI* and identification of cutting zones *ICZ*). On the basis of *CTQ* values determined, the cutting results of both cutting methods were compared. The analyses presented in this paper proved that the *CTQ* index can be effectively used to compare the results of a cutting process conducted using different methods and under different conditions. The developed *CTQ* index is a universal valuation tool, allowing for quantitative evaluation of features related to the technological quality of cutting process results.

## 1. Introduction

The operation of cutting is carried out on a very wide range of materials: from those used in technology (metals, plastics), through mineral raw materials and derivatives (rocks, concrete), to natural biological materials (wood, organic tissue: bones, muscles). Diversity of cut materials is related to the fact that they are used in many spheres of human activity: industry, medicine, services, etc. The diversity of properties of materials to be cut makes it necessary to use different methods and ways of cutting [1,2,3,4].

Cutting of metals is an essential technological operation, from which the entire process of manufacturing in the machine industry usually begins. The development of cutting methods is a response to the increasing demands of new materials and the desire to increase the quality and efficiency of processes, ranging from straight cutting, through 2D cutting (in two dimensions) to 3D cutting (in three dimensions) [5,6]. One of the most important groups of cutting operations used in industry today is cutting with concentrated energy: water jet or abrasive water jet (AWJ), laser, plasma, ultrasonic in vacuum, oxy-gas and electron beam cutting [7,8,9,10,11,12].

This paper focuses on a comparative analysis of the results of laser cutting (laser cutting) and abrasive water jet. These techniques have become popular in the last decade in industrial practice, which is also due to their versatility resulting from the numerical control of the cutting head motion path.

A high-pressure water jet is ideal for cutting different hardnesses of steel, ensuring an even and accurate cut [13]. A variation of water jet cutting is hydro-jet technology, which uses a concentrated stream of energy in the form of a high-pressure water jet containing admixtures of abrasive grains [14]. The locally applied large amount of accumulated energy (in the form of accelerated abrasive grains) causes effective erosion of the material due to the detachment of its microparticles from the basic mass of workpiece. In this process, the kinetic energy of the abrasive grains is converted into deformation energy of the material in the machining zone.

The advantages of this technique include: obtaining high quality cutting surface, no structural changes of the material at the edges (no heat affected zone), no thermal deformation, high surface cleanliness, short process preparation and finishing times and high process efficiency. This method is particularly profitable for small and medium series of products [3,4,5,14,15]. Water jet cutting using a highly compressed water jet allows cutting almost all soft materials such as insulation materials, cardboard, etc. When cutting hard materials such as metal, glass, stone, etc., an abrasive material is added to the water stream increasing the cutting force and at the same time making it possible to cut materials up to 300 mm thick. The main disadvantages of this technique include the tendency to cause delamination or visible striations on the surfaces subjected to the jet.

Laser cutting is a process in which the energy of the photon in a form of laser beam, continuous or pulsed, causes the melting of the cut material in the cutting gap or the simultaneous melting and vaporization and burning of the cut material: metals, some ceramics or thermoplastics.

The advantages of laser cutting include [12,16,17]:

- Easy automation of the process and its high flexibility,
- Wide range of materials that can be cut (from very soft and brittle to very hard),
- High speed of the process,
- Narrow cutting gap and heat affected zone,
- The possibility of obtaining semi-products on dimension and contactless process resulting in the lack of tool wear,
- High degree of process automation,
- The possibility of obtaining high precision cutting,
- Control of energy supply to the material to be cut,
- High process flexibility when changing the production profile, as there is no need to change cutting tools.

The laser cutting process is suitable for cutting mild steel up to about 30 mm thick. Obtaining good results above the 25 mm barrier requires high quality of all elements such as material (steel to be laser cut), gas purity, nozzle condition and beam quality. The laser cutting process requires a simultaneous, coaxial flow of gas with the laser beam. This gas is called assist gas. Its task is to blow out the resulting products (liquid and vaporized material) from the cutting gap.

The common features of both characterized techniques—high cutting performance, wide range of cut materials and relatively high cutting precision [12,16]—mean that the two methods can often be used interchangeably. As a result, in order to make a rational decision on the choice of cutting method, it is necessary to estimate the technological quality of this operation [16].

The term quality can be defined as the relationship to certain characteristics of a product or service that affect the customer’s perception of it. It is the degree to which a set of inherent properties satisfies the requirements. The assessment of quality depends, among other things, on experience, knowledge, and demand for the product. This concept is subject to change as a result of human development, product and quality changes. Quality is significantly influenced by the level of awareness of employees, superiors and the degree of implementation of quality concepts within the company. The quality level is also determined by the customers. Quality is a multidimensional and interdisciplinary concept. Due to the place of shaping the quality in the manufacturing process, we can distinguish technological quality, functional quality and operational quality of the product. Technological quality changes with the use of successive technological operations from the pre-treatment operation, through the shaping treatment to the finishing treatment operation. In the case when the product has a complex structure, the assembly operations have a significant influence on the shaped quality, as a result of which the functional quality is obtained. On the other hand, after the product is handed over to the customer, the operating quality can be distinguished, which is greatly affected by the conditions and intensity of operation [1,14,15,16,17,18,19,20].

Despite extensive knowledge of the cutting methods described, no universal method has been developed so far for evaluating the technological quality of elements shaped by different cutting processes. The aim of the research described in this article was to fill this gap and to propose the author’s methodology for the assessment of the technological quality of the surface of X5CRNI18-10 steel shaped as a result of laser cutting and abrasive water jet cutting. A synthetic index of technological quality assessment of the surface after cutting *CTQ* was proposed. On the basis of *CTQ* values determined, the cutting results of both methods were compared, taking into account in the evaluation the parameters of the surface texture, deviations of dimensions of cut elements and the results of microscopic observations of deformation of the formed cut surface.

The aim of the study was to determine the most advantageous parameters of the investigated processes with respect to the assessed indices of technological quality assessment: dimensional accuracy, surface texture and results of microscopic observations of surface deformations after cutting of sheet made of X5CrNi18-10 steel [21,22,23]. In order to achieve the goal, 54 elements were cut out, 27 of them by laser and 27 by abrasive water jet, at variable set values of the considered cutting processes.

## 2. Materials and Methods

### 2.1. Experimental Positions and Workpiece

**Figure 1:**
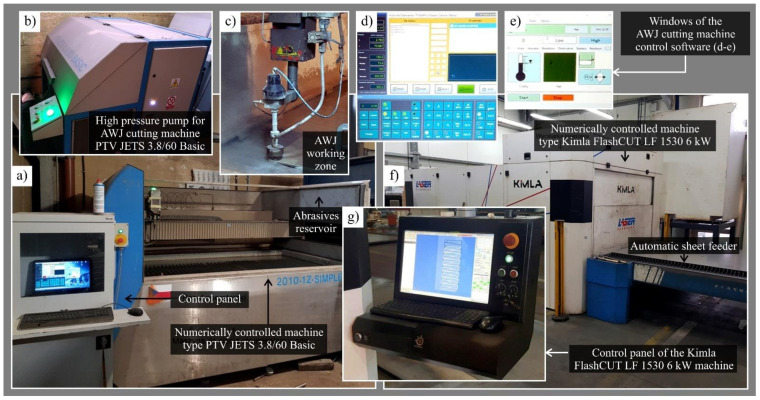
Experimental positions used in the study: (**a**) general view of numerically controlled AWJ cutting machine Kimla FlashCUT LF 1530 6 kW (Kimla, Częstochowa, Poland); (**b**) view of the high pressure pump for AWJ cutting machine; (**c**) view of the AWJ cutting head; (**d**,**e**) windows of the AWJ cutting machine control software; (**f**) general view of numerically controlled laser cutting machine type PTV JETS 3.8/60 Basic (PTV, Hostivice, Czech Republic); (**g**) view of the laser cutting machine control panel.

Laser cutting was carried out using numerically controlled Kimla FlashCUT LF 1530 6 kW (Kimla, Częstochowa, Poland)–Figure 1a–e [24]. Abrasive water jet cutting was carried out using a numerically controlled machine type PTV JETS 3.8/60 Basic (PTV, Hostivice, Czech Republic)–Figure 1f,g [25].

The cutting process was carried out starting from determining the position and fixing of the input material, which was a sheet made of X5CrNi18-10 steel with dimensions of 1250 mm × 2500 mm, from which workpieces of a rectangular shape 100 mm × 30 mm were cut. Then, the cutting process was programmed according to the adopted experiment plan and given unique designations.

### 2.2. Measurement of Process Results

In the next stage of the study, we proceeded to measure the dimensional accuracy of the machined workpieces. For this purpose, QLR MMT digital micrometers (Qinghai Measuring and Cutting Tools Group Company, Xining, China) with measuring ranges of 25–50/0.001 mm and 50–100/0.001 mm were used. Length and width measurements were made by measuring the distance between the opposite edges of the cut objects at three locations. Measurements of the geometric structure of the surface formed in the laser and abrasive water jet cutting processes were also carried out using the multi-head measuring system Talysurf CLI 2000 from Taylor-Hobson Ltd. (Leicester, UK) [26]. In addition, microscopic images of the intersection surface were recorded by opto-digital microscopy using a digital measuring microscope type Dino-Lite Edge AM7915MZT from ANMO Electronics Co. (Taiwan, China) [27]. The observations were made in three areas of the surface: the upper (area I), middle (area II) and lower (area III); the designations refer to the direction of the energy stream.

### 2.3. Process Parameters

Table 1 presents a summary of the inputs (fixed and constant) and outputs of the study.

The selected range of changes of the adjustable parameters of both cutting processes (given in Table 1) resulted from the technological capabilities of the applied test stands. Both the plate thickness *PT* and the cutting parameters were selected in such a way that it was possible to effectively cut the workpiece material within the entire range of variation of cutting conditions.

### 2.4. Synthetic Index of Cutting Technological Quality CTQ

Three groups of factors were taken into account in the assessment of technological quality of the surface after cutting: selected surface texture parameters (arithmetic mean deviation of the surface *Sa* and total height of the surface *St*), results of measurements of dimensional accuracy of cut elements (length deviation *LD* and width deviation *WD*) as well as indicators of surface morphology estimated on the basis of microscopic images of the surface after cutting (deformation intensity *DI* and identification of cutting zones *ICZ*). An attempt was made to correlate these factors with a relation that would synthetically allow to assess the technological quality of the shaped surface. On the basis of these assumptions, a synthetic index for the assessment of the technological quality of the surface after cutting *CTQ* was developed, determined by the Equation (1):

(1) $$\mathrm{CTQ}=\frac{3-\left[\left(Sa_{n}\cdot St_{n}\right)+\left|\left(LD_{n}\cdot WD_{n}\right)\right|+\left(DI_{n}\cdot ICZ_{n}\right)\right]}{3}\cdot 100\%,$$

where, subscripts *n* denote the values after normalization. Surface texture parameters (*Sa* and *St*) placed in the numerator of Equation (1) represent recognizable features of the solid (cut surface) associated with irregularities with relatively small vertex distances in 3D. Length and width deviation (*LD* and *WD*) are a measure of the difference of the actual dimension (determined by the measurement procedure) from the assumed (nominal) dimension, resulting from the values set in the cutting machine control program. On the other hand, deformation intensity *DI* and identification of cutting zones *ICZ* determine the features of surface morphology, which are characteristic for the cutting process. These features could be evaluated by qualitative analyses of microscopic images.

The construction of the proposed *CTQ* index (1) assumes dividing the numerator by 3 to obtain the resultant value from the range of 0–1 which, when multiplied by 100%, would allow expressing the technological quality of the evaluated surface after cutting with values from the range 0–100%. Due to the occurrence of negative values in the assessment of the dimensional accuracy, it was additionally necessary to express the product of the *LD* and *WD* deviations as an absolute value. As a result, *CTQ* index takes higher value for smaller sum of products, corresponding to smaller values of surface texture parameters, smaller dimensional deviations and smaller surface deformations after cutting.

## 3. Results and Discussion

The results of the conducted research were presented in four sections relating to surface texture analysis (Section 3.1), dimensional accuracy analysis (Section 3.2), microscopic image analysis (Section 3.3) and finally technological quality analysis (Section 3.4) workpieces machined using both cutting methods.

### 3.1. Surface Texture Analysis

The results of microtopography measurements of the cut surface showed significant differences between the values of selected surface texture parameters determined in the input and output areas of the abrasive water jet and the photon flux from the material. This proves reduced efficiency of water abrasive jet action as an erosive tool in shaping of the lower part of cutting zone. It is similar in the case of laser cutting, where the curvature of the laser beam in the lower part of the cut element distorts the cut edge from the middle of the plate thickness downwards. Therefore, it was decided that the measurements of microtopography of the cut surface will be made in the lower part of the cutting zone (at a distance of 1.0 mm from the bottom edge). The surface texture was recorded on each specimen at the bottom of the cutting zone with dimensions (*x*, *y* axis): 4.8 × 4.8 mm. During the measurement, 321 profiles (*y*-axis) were recorded. The distance between the profiles was 15 μm. On one profile 2401 points were registered (*x*-axis). The distance between profile points was 2 μm. Each measurement was performed in single-pass mode. The measurement time of one area was 4024 s. Data obtained during the measurements were analyzed using specialized software TalyMap Platinum 4.0 (Digital Surf, Besançon, France).

**Figure 2:**
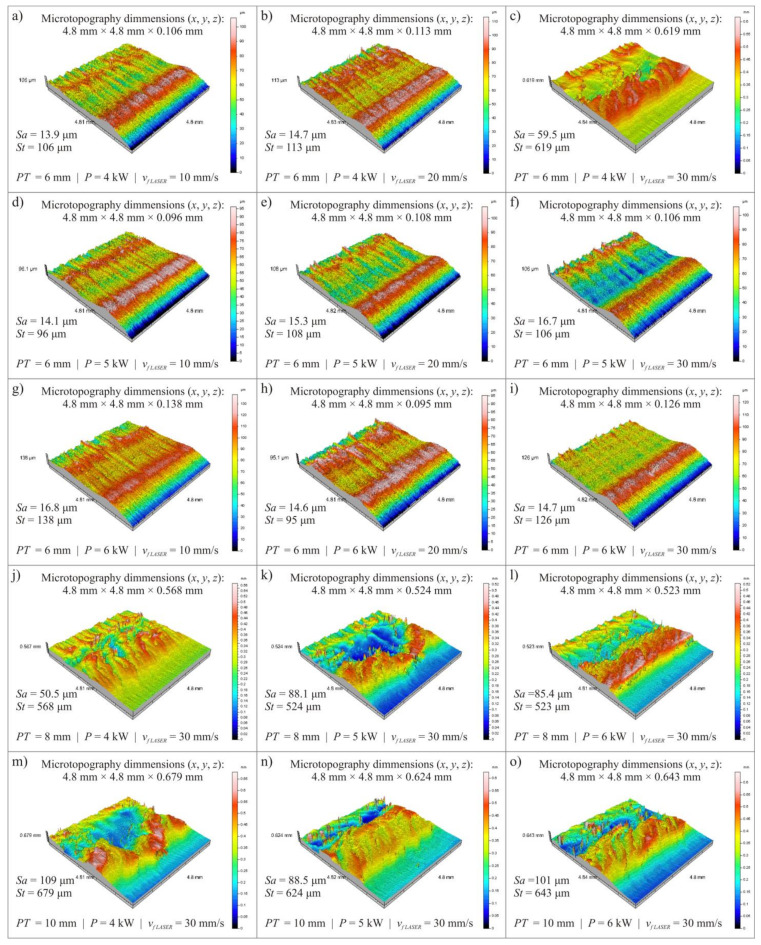
An overview of selected microtopographies of cut surfaces of elements cut by laser cutting: (**a**–**i**) *PT* = 6 mm, *P* = 4, 5, 6 kW, *v_f LASER_* = 10, 20, 30 mm/s; (**j**–**l**) *PT* = 8 mm, *P* = 4, 5, 6 kW, *v_f LASER_* = 30 mm/s; (**m**–**o**) *PT* = 10 mm, *P* = 4, 5, 6 kW, *v_f LASER_* = 30 mm/s.

**Figure 3:**
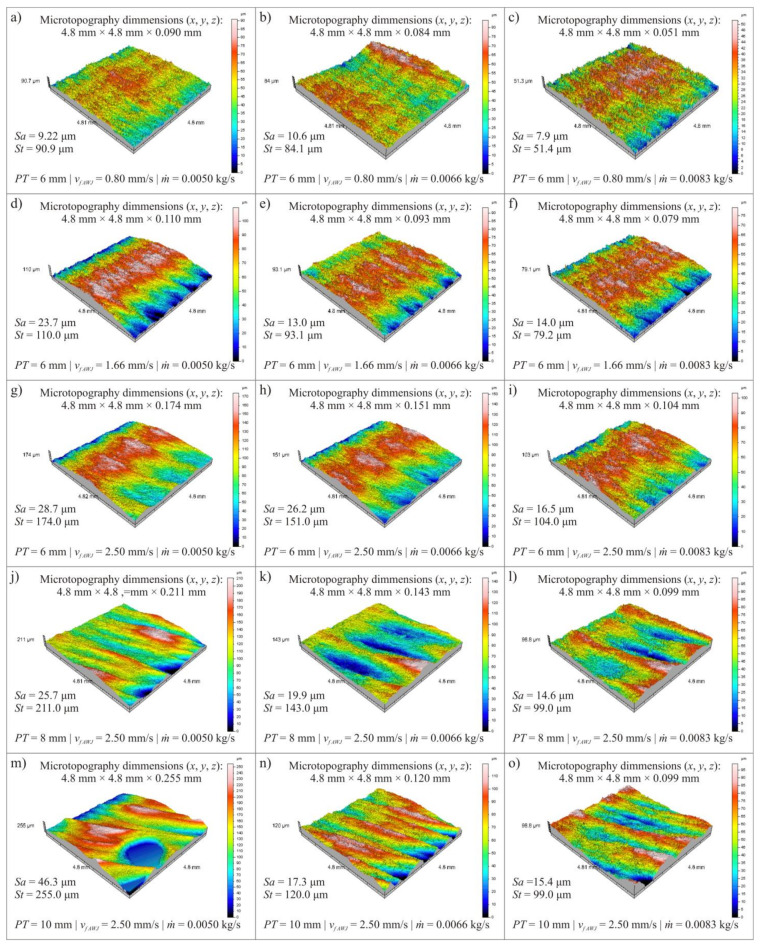
An overview of selected microtopographies of cut surfaces of elements cut by abrasive water jet: (**a**–**i**) *PT* = 6 mm, *v_f AWJ_* = 0.80; 1.66; 2.5 mm/s, *ṁ* = 0.0050, 0.0066, 0.0083 kg/s; (**j**–**l**) *PT* = 8 mm, *ṁ* = 0.0050, 0.0066, 0.0083 kg/s, *v_f AWJ_* = 2.50 mm/s; (**m**–**o**) *PT* = 10 mm, *ṁ* = 0.005, 0.0066, 0.0083 kg/s, *v_f AWJ_* = 2.50 mm/s.

An overview of the surface microtopography obtained is shown in Figure 2 for the surface after laser cutting process and in Figure 3 for the surface after abrasive water jet cutting process.

For each of the obtained surface microtopographies, the values of two surface texture parameters were determined:

- *Sa*–arithmetic mean deviation of the surface (μm),
- *St*–total height of the surface (μm).

Table 2 and Table 3 show the values of selected surface texture parameters after cutting the plate with thickness *PT* = 6, 8 and 10 mm for variable power *P* and cutting head feed rate *v_f LASER_* in laser cutting process as well as variable abrasive flow rate *ṁ* and cutting head feed rate *v_f AWJ_* in abrasive water jet cutting process.

The obtained results of surface texture measurements (Table 2 and Table 3) prove that the decrease in roughness amplitude parameter values, which can be interpreted as an increase in the quality of surfaces cut by the water-abrasive jet, is affected by decreasing the value of abrasive flow rate *ṁ* and increasing the cutting head feed speed *v_f_*_* AWJ*_. In the case of laser cutting an increase in the cutting head feed rate *v_f_*_* LASER*_ affects increase of surface texture amplitude parameters (*Sa* and *St*) of the cut surface. In the conducted study, the effect of laser cutting power *P* was inconclusive.

The determined values of *Sa* and *St* parameters prove that plate thickness *PT* for both cutting methods significantly affects the quality of the surface after cutting. At the smallest plate thickness *PT* = 6 mm in the examined range, no significant traces of the effect of concentrated energy flux were observed, which became apparent when cutting plate thickness *PT* = 8 and 10 mm, which increased the values of surface texture parameters.

### 3.2. Dimensional Accuracy Analysis

The dimensional accuracy measurements performed in the described study allowed determination of the dimensional deviations of the workpieces after cutting. The largest positive deviation of length *LD* = 0.609 mm was observed in the element cut from a plate of thickness *PT* = 8 mm by laser with power *P* = 4 kW and cutting head feed rate *v_f_*_* LASER*_ = 30 mm/s. On the other hand, the largest positive deviation of width *WD* = 0.563 mm was measured in the element cut out of the plate with thickness *PT* = 6 mm by the laser with power *P* = 4 kW and cutting head feed rate *v_f_*_* LASER*_ = 30 mm/s. The largest negative value of the length deviation *LD* = −0.236 mm was observed in the element cut from a sheet of thickness *PT* = 10 mm by laser with power *P* = 6 kW and cutting head feed rate *v_f_*_* LASER*_ = 10 mm/s. On the other hand, the largest negative deviation of the width *WD* = −0.237 mm was determined in the case of the workpiece cut from a plate of thickness *PT* = 10 mm by the laser with power *P* = 6 kW and cutting head feed rate *v_f_*_* LASER*_ = 10 mm/s. The analysis of the presented results of dimensional accuracy measurements of cut out elements showed that the lowest dimensional accuracy was obtained in the process of laser cutting. It should be noted that this process was carried out with about 10 times higher feed rate values of the cutting head in comparison with the abrasive water jet cutting process.

**Figure 4:**
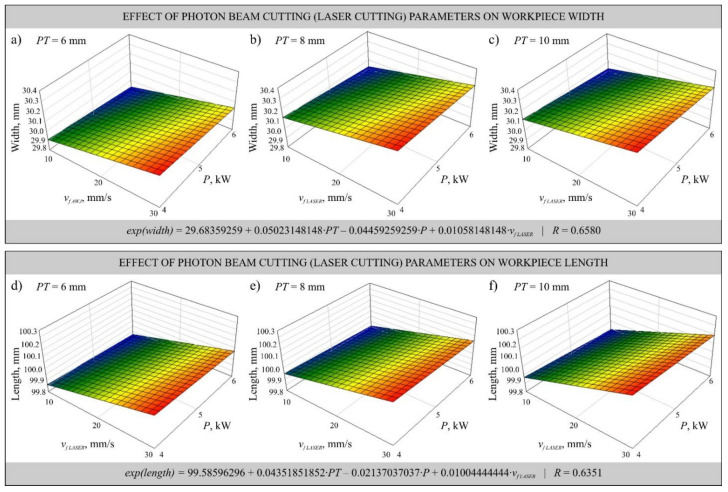
Mathematical models developed using data from measurements of the width (**a**–**c**) and length (**d**–**f**) of elements cut by laser beam from plate of thickness *PT* = 6 mm (**a**,**d**), *PT* = 8 mm (**b**,**e**) and *PT* = 10 mm (**c**,**f**) as a function of changes in the laser head feed rate *v_f LASER_* and laser power *P*.

**Figure 5:**
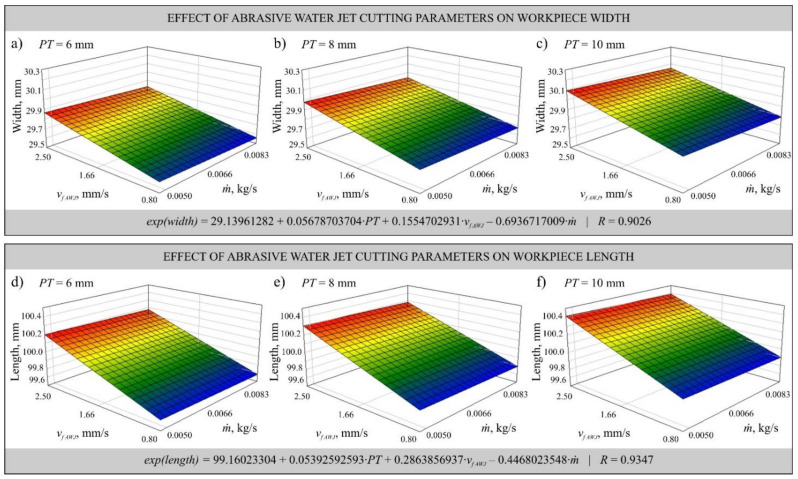
Mathematical models developed using data from measurements of the width (**a**–**c**) and length (**d**–**f**) of elements cut by laser beam from plate of thickness *PT* = 6 mm (**a**,**d**), *PT* = 8 mm (**b**,**e**) and *PT* = 10 mm (**c**,**f**) as a function of changes in the AWJ head feed rate *v_f AWJ_* and abrasive flow rate *ṁ*.

The obtained measurements allowed determination of the mathematical models that informed the most favorable (in the assumed range of variability) cutting parameters, i.e., laser power *P*, feed rate of both cutting heads *v_f_*_* LASER*_ and *v_f_*_* AWJ*_ and abrasive flow rate *ṁ*. Multivariate correlation coefficients *R* were determined for the models to assess model adequacy. The models were specified as first-order exponential functions without interaction and plotted as shown in Figure 4 and Figure 5.

The developed mathematical models describe the influence of changes of the laser head feed rate *v_f_*_* LASER*_ and power *P* during laser cutting (Figure 4) as well as *v_f_*_* AWJ*_ and abrasive flow rate *ṁ* (Figure 5) on the dimensional accuracy of the cut workpieces. In Figure 4, where the results are presented in the form of a graph of width *w* and length *l*, can be seen a slight effect of the laser power *P* on the dimensional accuracy, while the effect of the head feed rate *v_f_*_* LASER*_ causes deviations of up to 0.2 mm, which is particularly evident in Figure 4b,c. The plate thickness *PT* also affects the dimensional deviation. The thicker the plate, the larger the positive deviation. The effect of varying cutting parameters causes the phenomenon of post-treatment marks, which can also adversely affect the measurements.

In abrasive water jet cutting the trends are similar, however, the deviation occurring is larger and amounts even to 0.3 mm for width *w*, which can be seen in Figure 5b, and 1.0 mm for length *l*, which can be seen in Figure 5d–f. The obtained measurement results showed that increasing the head feed rate *v_f_*_*AWJ*_ causes deviation from perpendicularity of the surface formed in the abrasive water jet cutting process, which results in differences in the dimensions registered on the lower and upper parts of the cut workpieces. The abrasive flow rate *ṁ* slightly influenced the difference between the set dimension of the width *w* and the one actually cut out, which is visible in Figure 5a–c. However, no significant effect of abrasive flow rate *ṁ* on the deviation of length *l* was observed. This can be explained by the fact that the abrasive is only a factor that improves the cutting process, causing more degradation of the cut material, but not significantly affecting the dimension of the cut workpiece. Consequently, the inclination of the cutting plane is reduced.

### 3.3. Microscopic Image Analysis

**Figure 6:**
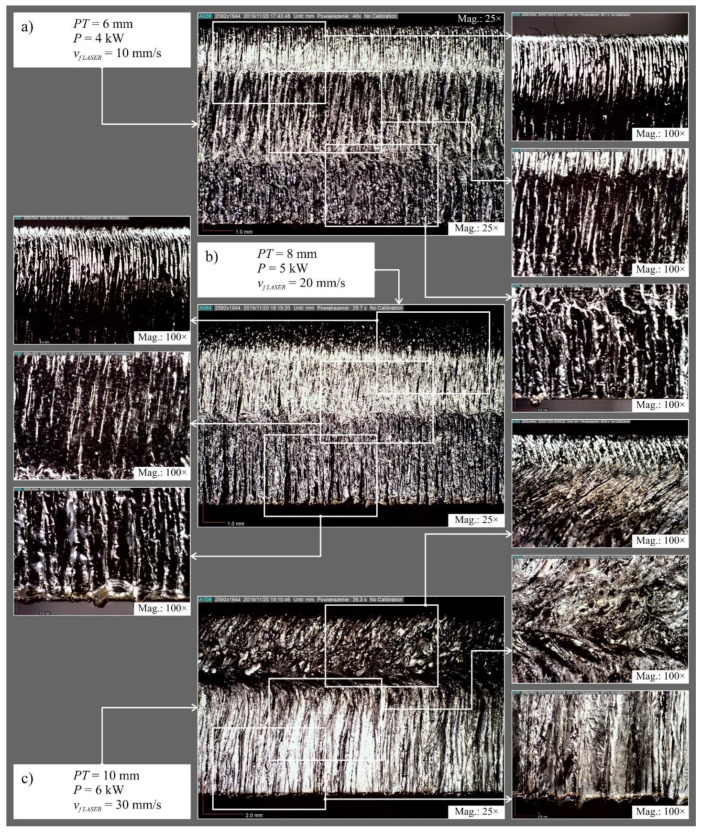
Examples of microscopic images of surfaces after laser cutting: (**a**) *PT* = 6 mm, *P* = 4 kW, *v_f_*_* LASER*_ = 10 mm/s; (**b**) *PT* = 8 mm, *P* = 5 kW, *v_f_*_* LASER*_ = 20 mm/s; (**c**) *PT* = 10 mm, *P* = 6 kW, *v_f_*_* LASER*_ = 30 mm/s.

**Figure 7:**
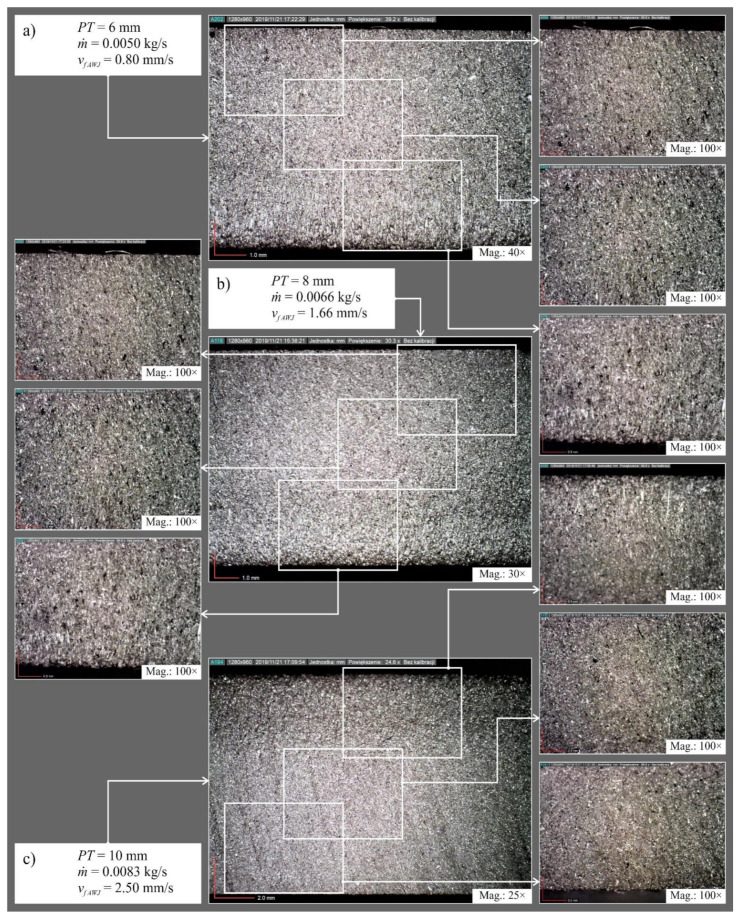
Examples of microscopic images of surfaces after abrasive water jet cutting: (**a**) *PT* = 6 mm, *ṁ* = 0.0050 kg/s, *v_f_*_* AWJ*_ = 0.80 mm/s; (**b**) *PT* = 8 mm, *ṁ* = 0.0066 kg/s, *v_f_*_* AWJ*_ = 1.66 mm/s; (**c**) *PT* = 10 mm, *ṁ* = 0.0083 kg/s, *v_f_*_* AWJ*_ = 2.50 mm/s.

Figure 6 shows examples of microscopic images of surfaces after laser cutting and Figure 7 shows examples of microscopic images of surfaces after abrasive water jet cutting.

Observation of the microscopic images allowed identification of the defects in the cut surface and to observe the quality zones present in both cutting methods. The occurrence of mechanical erosion of abrasive grains in abrasive water jet cutting noticeably affects the degradation of the cut surface. Increasing the plate thickness from *PT* = 6 to 8 mm and then to 10 mm resulted in an increase in the number of surface deformations observed by microscopy technique in both cutting methods. On the other hand, at the smallest value of abrasive flow rate *ṁ* on the surface after abrasive water jet cutting, clear deformation traces were recorded for plate thicknesses *PT* = 8 and 10 mm. A similar situation was observed on the surface after cutting elements shaped with the average and with the highest head feed ratio *v_f_*_* AWJ*_ = 1.66 and 2.50 mm/s, respectively.

The surface after laser cutting was characterized by significantly higher deformation intensity compared to the surface after abrasive water jet cutting. Increasing the value of laser power *P* and the feed rate of the head *v_f_*_* LASER*_ had an adverse effect on the shape of the surface after cutting. This showed a clear division into three zones of the cut surface. In zone one, the trace of a precise cut perpendicular to the surface of the plate is noticeable. Zone two is characterized by a wavier surface, which is the result of energy being stored here. In zone three, the surface quality is strongly affected by the molten material ejected by the associated gas in the upper zone.

### 3.4. Technological Quality Analysis

**Figure 8:**
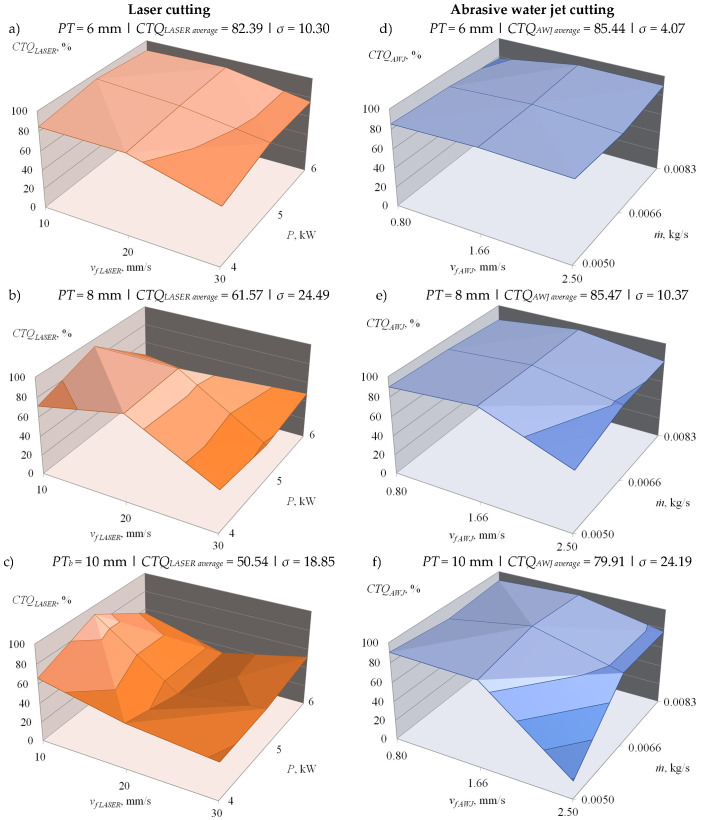
Changes in *CTQ* values of the surface after cutting plates with thicknesses *PT* of 6 mm (**a**,**d**), 8 mm (**b**,**e**) and 10 mm (**c**,**f**) depending on the variable power *P* and cutting head feed rate *v_f LASER_* in laser cutting process (**a**–**c**) as well as variable abrasive flow rate *ṁ* cutting head feed rate *v_f AWJ_* in abrasive water jet cutting process (**d**–**f**) (*σ*–standard deviation).

Table 4 and Table 5 present a summary of the values of the factors (before and after normalization) enabling determination of the synthetic index of technological surface quality assessment, respectively after laser cutting *CTQ_LASER_* (Table 4) and after abrasive water jet cutting *CTQ_AWJ_* (Table 5). The variability of the *CTQ* index values obtained is also presented in the form of graphs in Figure 8.

Analysis of *CTQ* index values (Table 4 and Table 5, Figure 8) allowed determination of the most advantageous parameters of both considered cutting processes from the point of view of technological quality assessment of workpieces after cutting. The evaluation took into account the selected surface texture parameters, deviations of dimensions of cut elements and microscopic observations of shaped cut surface deformation. As a result, it was determined that for the considered range of variability of the technological parameters of the laser cutting process, for particular plate thicknesses, the following parametric values can be regarded as the most advantageous:

- For sheet thickness *PT* = 6 mm: laser power *P* = 5 kW and working head feed rate *v_f_*_* LASER*_ = 10 mm/s (*CTQ_LASER_* = 93.85%),
- For sheet thickness *PT* = 8 mm: laser power *P* = 5 kW and working head feed rate *v_f_*_* LASER*_ = 10 mm/s (*CTQ_LASER_* = 92.31%),
- For sheet thickness *PT* = 10 mm: laser power *P* = 5 kW and working head feed rate *v_f_*_* LASER*_ = 10 mm/s (*CTQ_LASER_* = 90.90%).

However, for the abrasive water jet cutting process, the following parameter values were selected as the most favorable for the conditions under consideration:

- For sheet thickness *PT* = 6 mm: abrasive flow rate *ṁ* = 0.0083 kg/s and working head feed rate *v_f AWJ_* = 1.66 mm/s (*CTQ_AWJ_* = 90.68%),
- For sheet thickness *PT* = 8 mm: abrasive flow rate *ṁ* = 0.0050 kg/s and working head feed rate *v_f AWJ_* = 1.66 mm/s (*CTQ_AWJ_* = 96.30%),
- For sheet thickness *PT* = 10 mm: abrasive flow rate *ṁ* = 0.0066 kg/s and working head feed rate *v_f AWJ_* = 1.66 mm/s (*CTQ_AWJ_* = 99.41%).

On the example of analysis of the process of laser cutting and abrasive water jet cutting of stainless steel X5CRNI18-10 it was proved that the proposed synthetic index of technological quality assessment of the surface after cutting *CTQ* can be effectively used to determine the most favorable parameters of the cutting process. The design of the *CTQ* index allows taking into account other features of the analyzed surface and expanding their number, e.g., by adding parameters for evaluating the surface layer structure, surface microhardness, etc. The presented broad set of analyses showed that the developed *CTQ* index is a universal valuation tool, allowing for quantitative evaluation of features related to the technological quality of cutting process results.

## 4. Conclusions

The analyses presented in this paper in the scope of technological quality assessment of the workpieces after laser cutting and abrasive water jet cutting made it possible to formulate the following conclusions.

1. The obtained results of surface texture measurements of surfaces cut by the water-abrasive jet prove that the decrease in roughness amplitude parameter values (*Sa* and *St*) is affected by decreasing the value of abrasive flow rate *ṁ* and increasing the cutting head feed speed *v_f_*_* AWJ*_.
2. In the laser cutting process an increase in the cutting head feed rate *v_f_*_* LASER*_ affects increase of parameters *Sa* and *St* of the cut surface, at the same time the effect of laser cutting power *P* was inconclusive in this study.
3. For both analyzed cutting methods plate thickness *PT* significantly affects the quality of the surface after cutting–at the smallest plate thickness *PT* = 6 mm in the examined range, no significant traces of the effect of concentrated energy flux were observed, which became apparent when cutting plate thickness *PT* = 8 and 10 mm.
4. In the laser cutting process the dimensional accuracy of the cut workpieces affects mostly the head feed rate *v_f_*_* LASER*_ (high feed rate causes deviations of up to 0.2 mm) and plate thickness (the thicker the plate, the larger the positive deviation).
5. In abrasive water jet cutting trends are similar, however, the dimensional deviation occurring is larger and amounts even to 0.3 mm. Additionally it was noted that increasing the head feed rate *v_f_*_* AWJ*_ causes deviation from perpendicularity of the surface formed. No significant effect of abrasive flow rate *ṁ* on the dimensional deviation was observed.
6. Observation of microscopic images showed that increasing the plate thickness from *PT* = 6 to 8 mm and then to 10 mm resulted in an increase in the number of surface deformations in both cutting methods.
7. The surface after laser cutting was characterized by significantly higher deformation intensity (with visible division into three zones of the cut surface) compared to the surface after abrasive water jet cutting.
8. The analysis showed that the most favorable (highest) *CTQ* value of the entire set of analyzed results (*CTQ_AWJ_* = 99.41%) was obtained for the abrasive water jet cutting process with sheet thickness *PT* = 10 mm, abrasive flow rate *ṁ* = 0.0066 kg/s and working head feed rate *v_f AWJ_* = 1.66 mm/s.
9. The most favorable value of *CTQ* index for laser cutting process (*CTQ_LASER_* = 93.85%) was obtained for sheet thickness *PT* *=* 6 mm, laser power *P* = 5 kW and working head feed rate *v_f_*_* LASER*_ = 10 mm/s.
10. The relative (percentage) form of the *CTQ* index means that it can be used to compare the results of a cutting process conducted using different methods and under different conditions.

## Figures and Tables

**Table 1:** Specification of the input quantities (constant and variable) and output quantities of the tests conducted.

Cutting Process	Variable Input Quantities	Constant Input Quantities	Output Quantities
Laser cutting	Plate thickness *PT* in mm–is described as the thickness of a flat or coiled metallurgical product, much smaller than its length and width, the range used in testing is: 6, 8, 10 mm. Laser power *P* in kW–described as output power, normalized; it is a scalar physical quantity that reports the work done over time, the range used in the study is 4.0, 5.0, 6.0 kW. Cutting head feed rate *v_f LASER_* in mm/s–this is the feed rate of the cutting head relative to the workpiece per unit time, the range used in the study is 10, 20, 30 mm/s.	Cut material: X5CrNi18-10 steel. Constant distance of the laser lens from the plate to be cut of 1.5 mm. Lens diameter: 19.05 mm. The lens was positioned at the same consistent right angle to the cut plate throughout the cutting process.	Measurement results of overall dimensions (length *l* and width *w*) of machined workpieces. Results of surface texture parameter measurements after cutting using the following parameters: arithmetic mean deviation of the surface–*Sa*, total height of the surface–*St*. Results of qualitative analysis of microscopic images of cut surfaces.
Abrasive water jet cutting	Plate thickness *PT* in mm–the range used in testing is: 6, 8, 10 mm. Cutting head feed rate *v_f AWJ_* in mm/min–the range used in testing is: 0.80, 1.66, 2.50 mm/s. The process was controlled by adjusting the abrasive flow rate *ṁ* in kg/s–the range used in testing is: 0.005, 0.0066, 0.0083 kg/s.	Cut material: X5CrNi18-10 steel. Abrasive material: amaldine garnet mesh 80 with a nominal abrasive grit size of 0.18 mm in the range (106–300 µm). Fixed nozzle distance of 2 mm from the plate to be cut. Nozzle diameter: 0.25 mm. The nozzle was set at the same constant right angle to the plate throughout the cutting process. Water was supplied directly from the water supply system with a constant pressure of 0.3 MPa.	

**Table 2:** Values of the *Sa* parameter of the surface after cutting for plate with thickness *PT* = 6, 8 and 10 mm and for variable power *P* and cutting head feed rate *v_f LASER_* in laser cutting process as well as for variable abrasive flow rate *ṁ* and cutting head feed rate *v_f_*_* AWJ*_ in abrasive water jet cutting process.

Laser Cutting					Abrasive Water Jet Cutting				
Laser Power *P*, kW	Laser Head Feed Rate *v_f LASER_*, mm/s	*Sa* for *PT* = 6 mm, µm	*Sa* for *PT* = 8 mm, µm	*Sa* for *PT* = 10 mm, µm	AWJ Head Feed Rate *v_f AWJ_*, mm/s	Abrasive Flow Rate *ṁ*, kg/s	*Sa* for *PT* = 6 mm, µm	*Sa* for *PT* = 8 mm, µm	*Sa* for *PT* = 10 mm, µm
4.0	10	13.9	31.1	49.8	0.80	0.0050	9.22	10.0	7.49
4.0	20	14.7	25.5	51.0	0.80	0.0066	10.6	6.9	5.62
4.0	30	59.5	50.5	109.0	0.80	0.0083	7.9	6.33	5.56
5.0	10	14.1	25.4	19.7	1.66	0.0050	23.7	11.6	13.8
5.0	20	15.3	19.8	95.5	1.66	0.0066	13.0	8.86	7.15
5.0	30	16.7	88.1	88.5	1.66	0.0083	14.0	12.2	12.0
6.0	10	16.8	82.3	20.9	2.50	0.0050	28.7	25.7	46.3
6.0	20	14.6	88.2	84.5	2.50	0.0066	26.2	19.9	17.3
6.0	30	14.7	85.4	101.0	2.50	0.0083	16.5	14.6	15.4

**Table 3:** Values of the *St* parameter of the surface after cutting for plate with thickness *PT* = 6, 8 and 10 mm and for variable power *P* and cutting head feed rate *v_f LASER_* in laser cutting process as well as for variable abrasive flow rate *ṁ* and cutting head feed rate *v_f_*_* AWJ*_ in abrasive water jet cutting process.

Laser Cutting					Abrasive Water Jet Cutting				
Laser Power *P*, kW	Laser Head Feed Rate *v_f AWJ_*, mm/s	*St* for *PT* = 6 mm, µm	*St* for *PT* = 8 mm, µm	*St* for *PT* = 10 mm, µm	AWJ Head Feed Rate *v_f AWJ_*, mm/s	Abrasive Flow Rate *ṁ*, kg/s	*St* for *PT* = 6 mm, µm	*St* for *PT* = 8 mm, µm	*St* for *PT* = 10 mm, µm
4.0	10	106	151	453	0.80	0.0050	90.9	63.6	80.9
4.0	20	113	157	578	0.80	0.0066	84.1	46.7	39.6
4.0	30	619	568	680	0.80	0.0083	51.4	53.1	36.9
5.0	10	96	139	139	1.66	0.0050	110.0	87.7	79.2
5.0	20	108	126	767	1.66	0.0066	93.2	61.7	54.5
5.0	30	106	524	625	1.66	0.0083	79.2	79.2	91.2
6.0	10	138	639	151	2.50	0.0050	174.0	211.0	255.0
6.0	20	95	501	682	2.50	0.0066	151.0	143.0	120.0
6.0	30	126	523	643	2.50	0.0083	104.0	99.0	99.0

**Table 4:** List of the values of factors (before and after normalization) enabling the determination of cutting technological quality index after laser cutting *CTQ_LASER_* (subscripts *n* denote values after normalization).

Plate Thickness *PT*, mm	Laser Head Feed Rate *v_f LASER_,* mm/s	Laser Power *P*, kW	*Sa*, µm	*Sa_n_*	*St*, µm	*St_n_*	Average Dimensional Deviation				*DI*	*DI_n_*	*ICZ*	*ICZ_n_*	*CTQ_LASER_,* %
							*LD*, mm	*LD_n_*	*WD*, mm	*WD_n_*					
6	10	4.0	13.90	0.128	106.00	0.138	−0.005	−0.012	−0.086	−0.239	++	0.67	++	0.67	84.35
		5.0	14.10	0.129	96.00	0.125	−0.181	−0.437	−0.049	−0.136	+	0.33	+	0.33	93.85
		6.0	16.80	0.154	138.00	0.180	−0.138	−0.333	−0.210	−0.583	+	0.33	+	0.33	88.97
	20	4.0	14.70	0.135	113.0	0.147	−0.187	−0.452	−0.004	−0.011	++	0.67	++	0.67	84.21
		5.0	15.30	0.140	108.0	0.141	−0.031	−0.075	−0.018	−0.050	+++	1.00	+	0.33	88.22
		6.0	14.60	0.134	95.0	0.124	−0.166	−0.401	−0.041	−0.114	+	0.33	++	0.67	90.55
	30	4.0	59.50	0.546	619.0	0.807	0.173	0.418	0.348	0.967	+++	1.00	+	0.33	60.84
		5.0	16.70	0.153	106.0	0.138	−0.116	−0.280	−0.069	−0.192	++	0.67	+++	1.00	75.17
		6.0	14.70	0.135	126.0	0.164	−0.077	−0.186	−0.093	−0.258	++	0.67	+++	1.00	75.33
8	10	4.0	31.10	0.285	151.0	0.197	−0.144	−0.348	−0.141	−0.392	+++	1.00	++	0.67	71.25
		5.0	25.40	0.233	139.0	0.181	−0.114	−0.275	−0.104	−0.289	+	0.33	+	0.33	92.31
		6.0	82.30	0.755	639.0	0.833	0.056	0.135	0.040	0.111	+++	1.00	+++	1.00	45.20
	20	4.0	25.50	0.234	157.0	0.205	−0.182	−0.440	−0.128	−0.356	+	0.33	+	0.33	89.55
		5.0	19.80	0.182	126.0	0.164	−0.146	−0.353	−0.113	−0.314	+	0.33	+	0.33	91.68
		6.0	88.20	0.809	501.0	0.653	0.235	0.568	0.288	0.800	+++	1.00	+++	1.00	33.90
	30	4.0	50.50	0.463	568.0	0.741	0.414	1.000	0.239	0.664	+++	1.00	++	0.67	44.10
		5.0	88.10	0.808	524.0	0.683	0.279	0.674	0.336	0.933	+++	1.00	++	0.67	38.30
		6.0	85.40	0.783	523.0	0.682	0.248	0.599	0.217	0.603	+++	1.00	++	0.67	47.82
10	10	4.0	49.80	0.457	453.0	0.591	0.176	0.425	0.360	1.000	+	0.33	+++	1.00	65.84
		5.0	19.70	0.181	139.0	0.181	0.069	0.167	0.041	0.114	++	0.67	+	0.33	90.90
		6.0	20.90	0.192	151.0	0.197	−0.210	−0.507	−0.219	−0.608	+++	1.00	+++	1.00	55.13
	20	4.0	51.00	0.468	578.0	0.754	0.120	0.290	0.273	0.758	+++	1.00	+++	1.00	47.59
		5.0	95.50	0.876	767.0	1.000	0.113	0.273	0.278	0.772	+++	1.00	+++	1.00	30.44
		6.0	84.50	0.775	682.0	0.889	0.175	0.423	0.296	0.822	+++	1.00	+++	1.00	32.10
	30	4.0	109.00	1.000	680.0	0.887	0.167	0.403	0.244	0.678	+++	1.00	++	0.67	39.01
		5.0	88.50	0.812	625.0	0.815	0.205	0.495	0.293	0.814	+++	1.00	++	0.67	42.18
		6.0	101.00	0.927	643.0	0.838	0.025	0.060	0.022	0.061	+++	1.00	++	0.67	51.65

+++ very high | ++ high | + small

**Table 5:** List of the values of factors (before and after normalization) enabling the determination of cutting technological quality index after AWJ cutting *CTQ_AWJ_* (subscripts *n* denote values after normalization).

Plate Thickness *PT*, mm	AWJ Head Feed Rate *v_f AWJ_,* mm/s	Abrasive Flow Rate *ṁ*, kg/s	*Sa*, µm	*Sa_n_*	*St*, µm	*St_n_*	Average Dimensional Deviation				*DI*	*DI_n_*	*ICZ*	*ICZ_n_*	*CTQ_AWJ_,* %
							*LD*, mm	*LD_n_*	*WD*, mm	*WD_n_*					
6	0.8	0.0050	9.22	0.199	90.9	0.356	−0.191	−0.380	−0.294	−0.698	+	0.33	+	0.33	85.16
		0.0066	10.60	0.229	84.1	0.330	−0.207	−0.412	−0.321	−0.762	+	0.33	+	0.33	83.39
		0.0083	7.90	0.171	51.4	0.202	−0.298	−0.592	−0.372	−0.884	+	0.33	+	0.33	77.78
	1.66	0.0050	23.70	0.512	110.0	0.431	−0.027	−0.054	−0.374	−0.888	+	0.33	+	0.33	87.41
		0.0066	13.00	0.281	93.2	0.365	−0.081	−0.161	−0.421	−1.000	+	0.33	+	0.33	87.58
		0.0083	14.00	0.302	79.2	0.311	−0.045	−0.089	−0.363	−0.862	+	0.33	+	0.33	90.68
	2.5	0.0050	28.70	0.620	174.0	0.682	0.145	0.288	−0.106	−0.252	+	0.33	+	0.33	84.69
		0.0066	26.20	0.566	151.0	0.592	0.020	0.040	−0.216	−0.513	++	0.67	+	0.33	82.14
		0.0083	16.50	0.356	104.0	0.408	0.077	0.153	−0.193	−0.458	++	0.67	+	0.33	90.12
8	0.8	0.0050	10.00	0.216	63.6	0.249	−0.103	−0.205	−0.291	−0.691	+	0.33	+	0.33	89.85
		0.0066	6.90	0.149	46.7	0.183	−0.177	−0.352	−0.257	−0.610	+	0.33	+	0.33	88.30
		0.0083	6.33	0.137	53.1	0.208	−0.219	−0.435	−0.331	−0.786	+	0.33	+	0.33	84.02
	1.66	0.0050	11.60	0.251	87.7	0.344	0.170	0.338	−0.105	−0.249	+	0.33	+	0.33	96.30
		0.0066	8.86	0.191	61.7	0.242	−0.021	−0.042	−0.153	−0.363	+	0.33	+	0.33	94.32
		0.0083	12.20	0.263	79.2	0.311	−0.004	−0.008	−0.157	−0.373	+	0.33	+	0.33	93.54
	2.5	0.005	25.70	0.555	211.0	0.827	0.491	0.976	0.087	0.207	++	0.67	++	0.67	62.99
		0.0066	19.90	0.430	143.0	0.561	0.339	0.674	−0.028	−0.067	++	0.67	++	0.67	78.51
		0.0083	14.60	0.315	99.0	0.388	0.234	0.465	−0.012	−0.029	++	0.67	++	0.67	81.41
10	0.8	0.0050	7.49	0.162	80.9	0.317	−0.116	−0.231	−0.228	−0.542	+	0.33	+	0.33	90.49
		0.0066	5.62	0.121	39.6	0.155	−0.208	−0.414	−0.228	−0.542	++	0.67	+	0.33	84.52
		0.0083	5.56	0.120	36.9	0.145	−0.161	−0.320	−0.227	−0.539	+	0.33	+	0.33	90.04
	1.66	0.0050	13.80	0.298	79.2	0.311	0.195	0.388	0.031	0.074	++	0.67	+	0.33	88.59
		0.0066	7.15	0.154	54.5	0.214	0.179	0.356	−0.147	−0.349	+	0.33	+	0.33	99.41
		0.0083	12.00	0.259	91.2	0.358	0.050	0.099	−0.118	−0.280	+	0.33	+	0.33	94.20
	2.5	0.0050	46.30	1.000	255.0	1.000	0.442	0.879	0.212	0.504	+++	1.00	+++	1.00	18.57
		0.0066	17.30	0.374	120.0	0.471	0.503	1.000	0.033	0.078	++	0.67	++	0.67	76.58
		0.0083	15.40	0.333	99.0	0.388	0.451	0.897	0.056	0.133	++	0.67	++	0.67	76.76

+++ very high | ++ high | + small

## Nomenclature

AWJ	Abrasive water jet
*CTQ*	Cutting technological quality, –
*CTQ_LASER_*	Laser cutting technological quality, –
*CTQ_AWJ_*	Abrasive water jet cutting technological quality, –
*DI*	Deformation intensity, –
*DI_n_*	Normalized deformation intensity, –
*ICZ*	Identification of cutting zones, –
*ICZ_n_*	Normalized identification of cutting zones, –
*l*	Workpiece length, mm
*LD*	Length deviation, mm
*LD_n_*	Normalized length deviation, mm
*ṁ*	Abrasive flow rate, kg/s
*P*	Laser power, kW
*PT*	Plate thickness, mm
*R*	Multivariate correlation coefficient of mathematical model, –
*Sa*	Arithmetic mean deviation of the surface, μm
*Sa_n_*	Normalized arithmetic mean deviation of the surface, μm
*St*	Total height of the surface, μm
*St_n_*	Normalized total height of the surface, μm
*v_f LASER_*	Laser head feed rate, mm/s
*v_f AWJ_*	Abrasive water jet head feed rate, mm/s
*w*	Workpiece width, mm
*WD*	Width deviation, mm
*WD_n_*	Normalized width deviation, mm
